# Dietary *Enterococcus faecalis* SZ07 Improves Intestinal Health, Immune Function and Gut Microbiota Homeostasis in Golden Pompano (*Trachinotus ovatus*)

**DOI:** 10.3390/ani16213506

**Published:** 2026-11-06

**Authors:** Xiao Zhang, Jia Cai, Liqun Wang, Pengfei Li, Yi Li, Yu Huang, Dapeng Yu, Bei Wang, Jichang Jian, Huapu Chen, Sen Li

**Affiliations:** 1 Guangdong Key Laboratory of Aquatic Animal Disease Control and Healthy Culture, Guangdong Key Laboratory of Control for Diseases of Aquatic Economic Animals, Key Laboratory of Marine Ecology and Aquaculture Environment of Zhanjiang, Southern Marine Science and Engineering Guangdong Laboratory (Zhanjiang), College of Fishery, Guangdong Ocean University, Zhanjiang 524088, China; 2 Shenzhen Institute of Guangdong Ocean University, Guangdong Provincial Engineering Research Center for Aquatic Animal Health Assessment, Shenzhen Public Service Platform for Evaluation of Marine Economic Animal Seedings, Shenzhen 518120, China; 3 Guangxi Key Laboratory of Aquatic Biotechnology and Modern Ecological Aquaculture, Guangxi Academy of Sciences, Nanning 530007, China; 4 Guangdong Havwii Agricultural Group Co., Ltd., Zhanjiang 524088, China

**Keywords:** golden pompano, *Enterococcus faecalis*, probiotics, intestinal health, immunity, gut microbiota

## Abstract

*Trachinotus ovatus* (golden pompano) represents one of the dominant high-value mariculture fish species farmed across coastal regions of China. In intensive farming modes, intestinal damage and immune suppression are frequent issues in golden pompano; the widespread application of antibiotics in disease control has also triggered increasing worries about ecological risks and aquatic product safety. Host-origin probiotics have been widely recognized as a green and safe strategy to mitigate health risks and promote growth in farmed fish. Nevertheless, the regulatory roles and mechanisms of autochthonous *E. faecalis* strains on the intestinal health of golden pompano are still poorly understood. In this study, a 42-day feeding trial was conducted using golden pompano reared in a large-capacity floating net-cage system. The experimental fish were fed diets supplemented with different concentrations of *E. faecalis* SZ07 (1 × 10^5^ to 1 × 10^9^ CFU/g feed). Dietary supplementation with SZ07 at suitable doses (1 × 10^7^ to 1 × 10^8^ CFU/g feed) notably improved intestinal tissue structure, elevated the activities of digestive, antioxidant, and immune enzymes, and modulated the mRNA levels of key immune genes in the spleen. Analysis of the gut microbiota revealed that dietary *E. faecalis* SZ07 reshaped the intestinal microbial community, with an increased abundance of beneficial taxa and a decreased proportion of opportunistic pathogens. Taken together, our results indicate that autochthonous *E. faecalis* SZ07 acts as an effective probiotic to enhance intestinal health and immune function of golden pompano and offers technical support for the healthy development of its aquaculture industry.

## Simple Summary

Golden pompano is one of the most valuable marine-farmed fish in China. Intensive farming often leads to intestinal disorders and poor disease resistance in fish, while overuse of antibiotics brings risks for aquatic product safety and the surrounding marine environment. Probiotics isolated from the target fish itself may serve as safe alternatives to antibiotics for aquaculture production. In this work, we fed juvenile golden pompano with feed supplemented with different doses of host-origin *Enterococcus faecalis* SZ07 over 42 days. Suitable addition of this bacterial strain improved intestinal tissue structure, boosted digestive and antioxidant capacity, balanced immune responses, and optimized intestinal bacterial composition. The dose range of 1 × 10^7^–1 × 10^8^ CFU per gram of feed produced the most consistent beneficial effects. Our findings provide practical support for developing safe feed additives to promote healthy golden pompano farming.

## 1. Introduction

Golden pompano (*Trachinotus ovatus*) is a high-value mariculture species with increasing market demand in China [1]. This species exhibits persistent, fast group-swimming activity; insufficient swimming space within overly small confined enclosures can restrict its natural locomotion and induce stress responses, potentially altering physiological and immune functions [2]. For juvenile golden pompano reared in offshore floating net cages, stocking densities ≥ 100 fish/m^3^ may induce crowding stress, and lower densities are conducive to reducing confinement-related stress [3]. Under commercial mariculture circumstances, intensively cage-reared golden pompano frequently develop intestinal dysfunction, impaired digestive capacity, and suppressed non-specific immunity, which limits farming productivity [4]. Excessive antibiotic application in aquaculture has given rise to a series of issues, including antimicrobial resistance and ecological pollution, creating an urgent need to develop eco-friendly and safe feed additives [5]. The detrimental impacts of antibiotics on fish health have been documented across multiple species. For instance, dietary florfenicol (10–30 mg/kg for 30 days) induced severe intestinal histopathological damage, including epithelial cell degeneration and necrosis, in Nile tilapia (*Oreochromis niloticus*) [6]. Additionally, oxytetracycline treatment exerts an inhibitory effect on innate immunity. In gilthead seabream (*Sparus aurata*), it lowers hemolytic complement activity; in the Atlantic salmon (*Salmo salar*) SHK-1 cell line, it weakens phagocytic function [7,8]. In zebrafish (*Danio rerio*), gut microbiota disturbance caused by florfenicol failed to recover within the standard 15-day withdrawal period and significantly elevated the risk of subsequent *Aeromonas hydrophila* infection [9]. In rainbow trout (*Oncorhynchus mykiss*), oxytetracycline administration significantly elevated lipid peroxidation levels while reducing catalase and superoxide dismutase activities, indicating severe oxidative stress [10]. Collectively, these findings highlight that excessive or non-therapeutic antibiotic use poses substantial risks to fish intestinal health, immunity, and overall physiological homeostasis.

Aquaculture faces numerous threats from unrestricted antibiotic usage, which has driven broad adoption of probiotic additives. These biological supplements can optimize growth traits, intestinal integrity, innate immunity, and gut microbial balance in cultured fish [11]. Host-derived probiotics exhibit better adaptability and colonization ability in the host gut, showing more stable probiotic effects [12]. In Pacific white shrimp (*Penaeus vannamei*), dietary *Bacillus subtilis* supplementation increased the abundance of Proteobacteria and Firmicutes while improving growth performance [13]. It has been demonstrated that dietary *Lactobacillus plantarum* improves antioxidant capacity, innate immunity, and disease resistance against *Vibrio parahaemolyticus* in Manila clam (*Ruditapes philippinarum*) [14]. In European sea bass (*Dicentrarchus labrax*), dietary supplementation of live yeast *Saccharomyces cerevisiae boulardii* reduced the mRNA expression of intestinal pro-inflammatory cytokines, which indicates an anti-inflammatory function [15]. In golden pompano, the host-origin *B. subtilis* strain HGCC-1 enhanced growth performance and mitigated hepatic steatosis via regulating intestinal microbiota [16]. A recent comprehensive review identified Bacillus and Lactobacillus as the most studied probiotic genera for aquaculture applications [17].

As a representative lactic acid bacterium, certain strains of *E. faecalis* have been widely confirmed to possess favorable probiotic properties [18]. In Nile tilapia (*Oreochromis niloticus*), the combined dietary addition of *E. faecalis* and *Bacillus subtilis* effectively enhanced growth performance, immune function, and disease resistance to *Streptococcus agalactiae* challenge [19]. In snakehead fish (*Channa striatus*), *E. faecalis* isolated from the host intestine demonstrated strong probiotic potential against pathogenic infections [20]. In our earlier work, an *E. faecalis* strain, named SZ07, was isolated from the gut of healthy golden pompano, and its basic biological traits were preliminarily identified [21]. Nevertheless, research on the application of this autochthonous *E. faecalis* strain in golden pompano farming is still limited; specifically, its effects on intestinal structure, digestive enzyme activities, antioxidant capacity, immune responses, and gut microbiota composition have not been systematically elucidated.

Nevertheless, *E. faecalis* constitutes a genetically heterogeneous species; some isolates act as opportunistic pathogens harboring key virulence determinants and horizontally transferable antimicrobial-resistance genes, which raises safety concerns for feed-additive usage [22]. Our strain SZ07 was previously subjected to whole-genome and phenotypic biosafety assessment. Although several adhesion-related genes were annotated in its genome, key cytotoxic virulence gene *cylA* was absent. Even though a small set of resistance-related genes was detected, SZ07 remained sensitive to vancomycin, supporting its preliminary safety as a candidate aquaculture probiotic [23].

Given this research gap, we performed the present experiment in large-capacity floating net cages to investigate the regulatory roles of dietary *E. faecalis* SZ07 administration in golden pompano. Our results are expected to offer a reliable theoretical basis and practical technical support for developing *E. faecalis* SZ07 as a species-specific probiotic for the intensive farming of golden pompano.

## 2. Materials and Methods

### 2.1. Experimental Design

**Figure 1:**
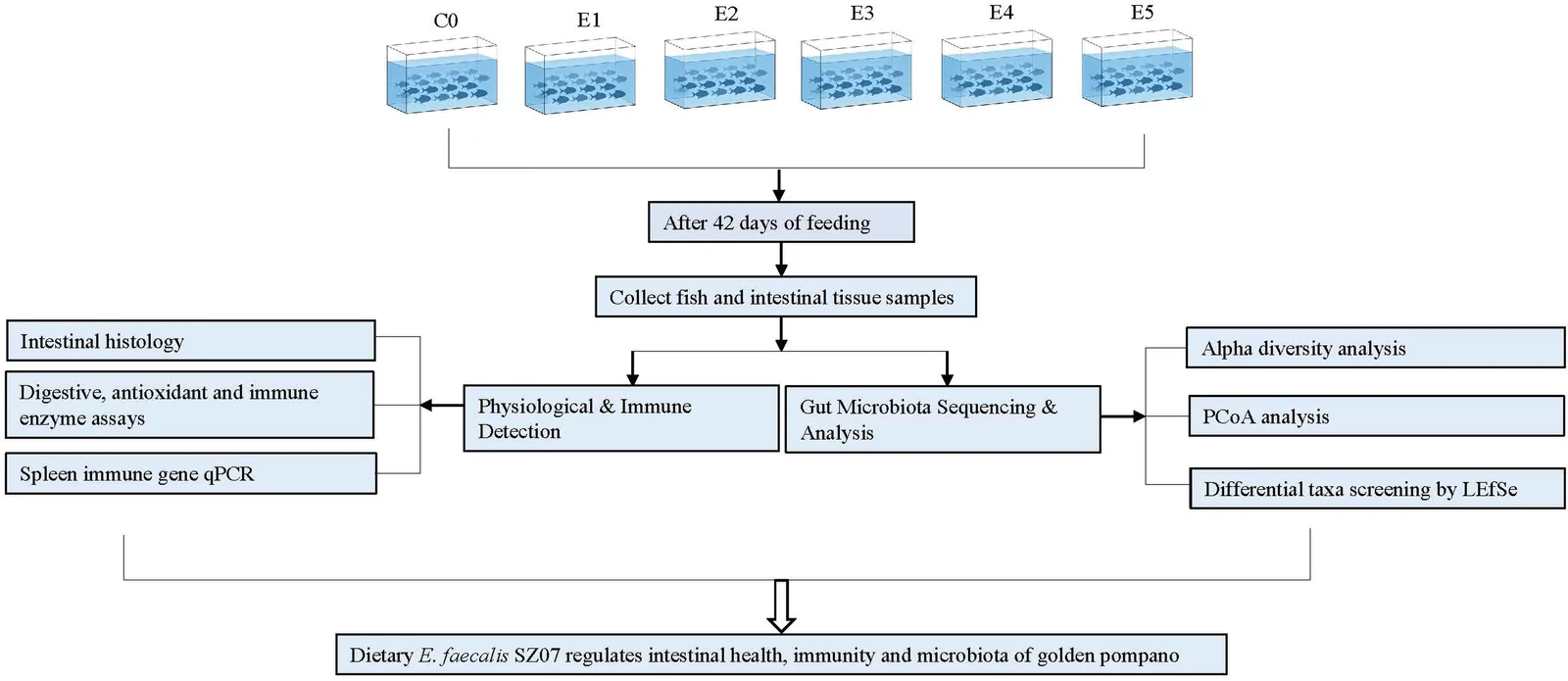
Schematic diagram of the experimental workflow.

To explore the impacts of dietary *E. faecalis* SZ07 on intestinal health and immune function of golden pompano, a 42-day feeding trial was performed in the current work. The detailed experimental workflow is illustrated in Figure 1. Briefly, gradient concentrations of *E. faecalis* SZ07 (10^5^–10^9^ CFU/g diet) were prepared and sprayed onto basal feed to produce experimental diets. All fish were randomly assigned to six groups and fed twice per day (08:00 and 16:00 h) over the 42-day trial period. After the feeding trial, tissue samples were collected for intestinal histology, biochemical and immune index detection, and gut microbiota sequencing.

The feeding trial was conducted in Zhanjiang, Guangdong, China (N 20°50′, E 109°42′). The feeding experiment ran from 6 June 2024 to 17 July 2024, lasting for 42 consecutive days. Each floating net cage was 2.0 m × 2.0 m × 1.5 m with a mesh size of 10 mesh. The water depth inside the culture pool was maintained at 1.2 m. During the experiment, water temperature fluctuated between 27.0 °C and 30.0 °C, while salinity was maintained within the range of 24–28 ppt throughout the trial.

This trial was arranged to evaluate how dietary supplementation with *E. faecalis* SZ07 affects intestinal health and innate immune performance of golden pompano. Six distinct treatments were set up in total: one blank control group and five treatment groups receiving gradient doses of the probiotic strain.

### 2.2. Probiotic Strain and Feed Preparation

The *E. faecalis* SZ07 strain used in this study was originally isolated from the intestinal tract of healthy golden pompano in our laboratory. Its basic biological characteristics have been reported in our previous study [21]. In addition, a genomic study focusing on its probiotic potential, biosafety, and aquaculture applicability has also been published [23]. The strain was preserved at −80 °C in MRS broth containing 50% (*v*/*v*) glycerol until use. SZ07 was cultured in MRS broth at 37 °C to mid-logarithmic phase. A linear regression model between OD_600_ and viable counts was calibrated by plate enumeration: y = 2947x − 717.4 (R^2^ = 0.9910), where y is bacterial concentration (10^6^ CFU/mL) and x is OD_600_; an OD_600_ value of 1 corresponds to approximately 2 × 10^9^ CFU/mL. The bacterial suspension was evenly sprayed onto the surface of feed pellets to achieve uniform distribution of the strain.

A commercial basal diet for marine fish (Hengxing Feed Co., Ltd., Zhanjiang, China) was used as the control diet. The proximate composition of the basal diet was as follows: crude protein 45%, crude fiber 4%, crude fat 7%, crude ash 15%, lysine 2.2%, total phosphorus 0.8%, and moisture 12%. The basal commercial feed had a pellet size of 3.4 mm; its main protein sources were fishmeal and soybean meal. Fish were fed to apparent satiation twice daily. Feed consumption per cage was not quantified in the present trial. Six experimental diets were prepared by spraying different concentrations of SZ07 bacterial suspension onto the surface of the basal diet using a gradient inoculation method. The final concentrations of SZ07 in the diets were 0 (control group, C0), 1 × 10^5^ (E1), 1 × 10^6^ (E2), 1 × 10^7^ (E3), 1 × 10^8^ (E4), and 1 × 10^9^ (E5) CFU/g diet, respectively. This 10-fold dose gradient covering 10^5^–10^9^ CFU/g was designed according to the dosage range reported for dietary Enterococcus faecalis in fish and our preliminary in vitro assays of strain SZ07 [24]. All prepared diets were air-dried in a ventilated place, sealed in plastic bags, labeled, and stored at 4 °C. Fresh diets were prepared every 7 days to ensure the viability of the probiotic.

### 2.3. Experimental Fish and Feeding Management

The experiment was conducted at a commercial aquaculture facility in Zhanjiang, Guangdong, China. Juvenile golden pompano were procured from a local seedling farm in Zhanjiang, Guangdong, for this investigation. The juvenile golden pompano were acclimated to experimental conditions for two weeks. After acclimation, the experimental fish with an initial body weight of 13.00 ± 0.95 g were randomly allocated into six floating net cages after the two-week acclimation period. Each cage contained 60 fish, giving a total of 360 experimental fish across all treatments. Randomization was performed by simple random sampling of individuals from the common acclimation stock pool. Each floating net cage had a volume of 6 m^3^. It should be noted that each dietary treatment occupied one independent net cage. The six dietary treatments were randomly arranged to minimize the potential positional effect.

At the end of the feeding trial, fish were fasted for 24 h before dissection and were randomly sampled from each independent net cage, with only one separate net cage allocated to each dietary treatment. Sampling was performed at 08:00. Fish were anesthetized with eugenol (50 mg L^−1^). All tissue samples for different assays were collected from distinct individual fish. Six individual golden pompano were harvested as biological replicates for each detection item. Midgut tissues from six fish were collected and fixed for histological observation; another six individuals were sampled to measure intestinal and hepatic biochemical enzyme activities as well as splenic immune gene expression via qPCR; and an extra six fish were obtained for intestinal gut microbiota high-throughput sequencing. All dissected tissue samples, except those reserved for histological observation, were instantly snap-frozen in liquid nitrogen and preserved at −80 °C for subsequent laboratory analyses.

### 2.4. Intestinal Histological Analysis

Intestinal tissue specimens were fixed with 4% paraformaldehyde solution for 24 h. Following fixation, samples were processed through gradient ethanol dehydration, xylene clearing, and paraffin wax embedding. Serial sections were cut, deparaffinized, rehydrated, and stained with hematoxylin and eosin (HE). The stained sections were mounted with neutral balsam and observed under a Zeiss Axioscope 5 light microscope (Carl Zeiss, Oberkochen, Germany). Intestinal morphometric parameters were quantified using Image-Pro Plus 6.0 (Media Cybernetics, Rockville, MD, USA).

For each intestinal tissue section, three morphological parameters were quantified: mucosal fold height, mucosal fold width, and muscle layer thickness. Mucosal fold height referred to the vertical distance from the mucosal fold base to its apical tip. Mucosal fold width was defined as the maximum transverse diameter perpendicular to the mucosal fold height axis. Muscle layer thickness corresponded to the thickness of the smooth muscle layer in the intestinal wall.

### 2.5. Biochemical Parameter Determination

Intestinal and liver tissue samples were homogenized in ice-cold phosphate-buffered saline. The homogenates were centrifuged, and the supernatants were collected for enzyme activity assays.

The activities of superoxide dismutase (SOD), catalase (CAT), glutathione peroxidase (GSH-Px), acid phosphatase (ACP), alkaline phosphatase (ALP), trypsin, amylase, and lipase were determined using commercial assay kits (Jin Shaoyuan Biotechnology Co., Ltd., Shanghai, China). All assays were performed strictly following the manufacturer’s instructions. Total protein was determined via the BCA method, and all enzyme activities were normalized to total protein concentration.

### 2.6. Immune-Related Gene Expression Analysis in Spleen

Total RNA was isolated from golden pompano spleen samples with Transzol Up Plus RNA Kit (TransGen Biotech Co., Ltd. Beijing, China) following the official kit operating protocols. RNA purity and concentration were detected via spectrophotometry, and 1% agarose gel electrophoresis was applied to confirm intact RNA bands.

EasyScript^®^ One-step gDNA Removal and cDNA Synthesis Super Mix (TransGen Biotech Co., Ltd.) was used to reverse transcribe qualified RNA into first-strand cDNA. All cDNA samples were preserved at −80 °C for subsequent qPCR detection. Quantitative real-time PCR amplification was conducted with the PerfectStart™ Green qPCR SuperMix kit (TransGen Biotech Co., Ltd.). The total reaction volume was 10 μL, containing 5.0 μL PerfectStart Green qPCR SuperMix, 1.0 μL forward primer, 1.0 μL reverse primer, 1.0 μL cDNA template, and 2.0 μL ddH_2_O. The cycling protocol was as follows: initial denaturation at 95 °C for 30 s, followed by 40 cycles of 95 °C for 5 s and 60 °C for 20 s. Melting curve analysis was carried out at 95 °C for 15 s, 60 °C for 1 min, and 95 °C for 1 s to confirm the specificity of amplification. All specific primer pairs were custom synthesized by Sangon Biotech Co., Ltd. (Shanghai, China), and full primer sequences are provided in Table 1. The 2^−ΔΔCt^ algorithm was adopted to calculate relative gene expression, with β-actin selected as the housekeeping reference gene, consistent with the methodology described in previous studies on golden pompano [25].

### 2.7. Intestinal Microbiota Sequencing Analysis

Microbial genomic DNA was extracted from golden pompano intestinal tissue samples with the MagPure Soil DNA LQ Kit (Magen Biotechnology Co., Ltd., Guangzhou, China) under standard kit protocols. DNA integrity and concentration were jointly evaluated through agarose gel electrophoresis and NanoDrop 2000 spectrophotometer detection (Thermo Fisher Scientific, Waltham, MA, USA). The V3–V4 hypervariable region of the bacterial 16S rRNA gene was amplified via PCR with barcoded primers 343F (5′-TACGGRAGGCAGCAG-3′)/798R (5′-AGGGTATCTAATCCT-3′) and high-fidelity DNA polymerase (Takara Bio Inc., Kusatsu, Shiga, Japan). AMPure XP magnetic beads (Beckman Coulter, Brea, CA, USA) were used to purify all PCR amplicons. Index amplification was carried out to build sequencing libraries, which were quantified by a Qubit fluorometer (Thermo Fisher Scientific, Waltham, MA, USA) and normalized for concentration. Paired-end sequencing was conducted on the Illumina NovaSeq 6000 platform (Illumina Inc., San Diego, CA, USA), with all sequencing services completed by OE Biotech Co., Ltd., (Shanghai, China). Cutadapt software (v4.0) was used to trim raw sequencing reads and remove adapter sequences. The DADA2 plug-in integrated into QIIME 2 (default parameters) was adopted to generate amplicon sequence variants (ASVs). Taxonomic classification of ASVs was completed against the SILVA 138 database via the q2-feature-classifier tool. Alpha diversity (Ace, Chao1, Shannon, Simpson indices) was analyzed in QIIME 2.

### 2.8. Statistical Analysis

All measurement data are expressed as mean ± standard deviation, with six independent fish as biological replicates within each single culture cage (*n* = 6 per treatment for all assays). SPSS 22.0 software (IBM, Armonk, NY, USA) was adopted for all statistical comparisons. Prior to one-way ANOVA, the Shapiro–Wilk test and Levene’s test were used to assess normality and homogeneity of variance, respectively. Appropriate data transformation was performed for datasets that failed to meet the assumptions of ANOVA. One-way ANOVA combined with Duncan’s multiple range test was applied to identify significant inter-group differences. Differences with *p* < 0.05 were defined as statistically significant. All visualization graphs were plotted with GraphPad Prism 8.0 (GraphPad Software, San Diego, CA, USA). For gut microbiota analysis, the Kruskal–Wallis test was used for comparisons of alpha-diversity indices. For beta diversity, principal coordinates analysis (PCoA) based on binary Jaccard distance was plotted in R, and Linear Discriminant Analysis Effect Size (LEfSe) with the built-in FDR multiple testing correction was applied to identify differentially abundant taxa. PCoA was visualized using the ggplot2 R package (v3.4.4); LEfSe analysis was performed within the QIIME2 plugin. Biomarker taxa were identified using LEfSe, with the Kruskal–Wallis test significance alpha set at 0.05 and the LDA score threshold of 2.0.

## 3. Results

### 3.1. Intestinal Morphology

**Figure 2:**
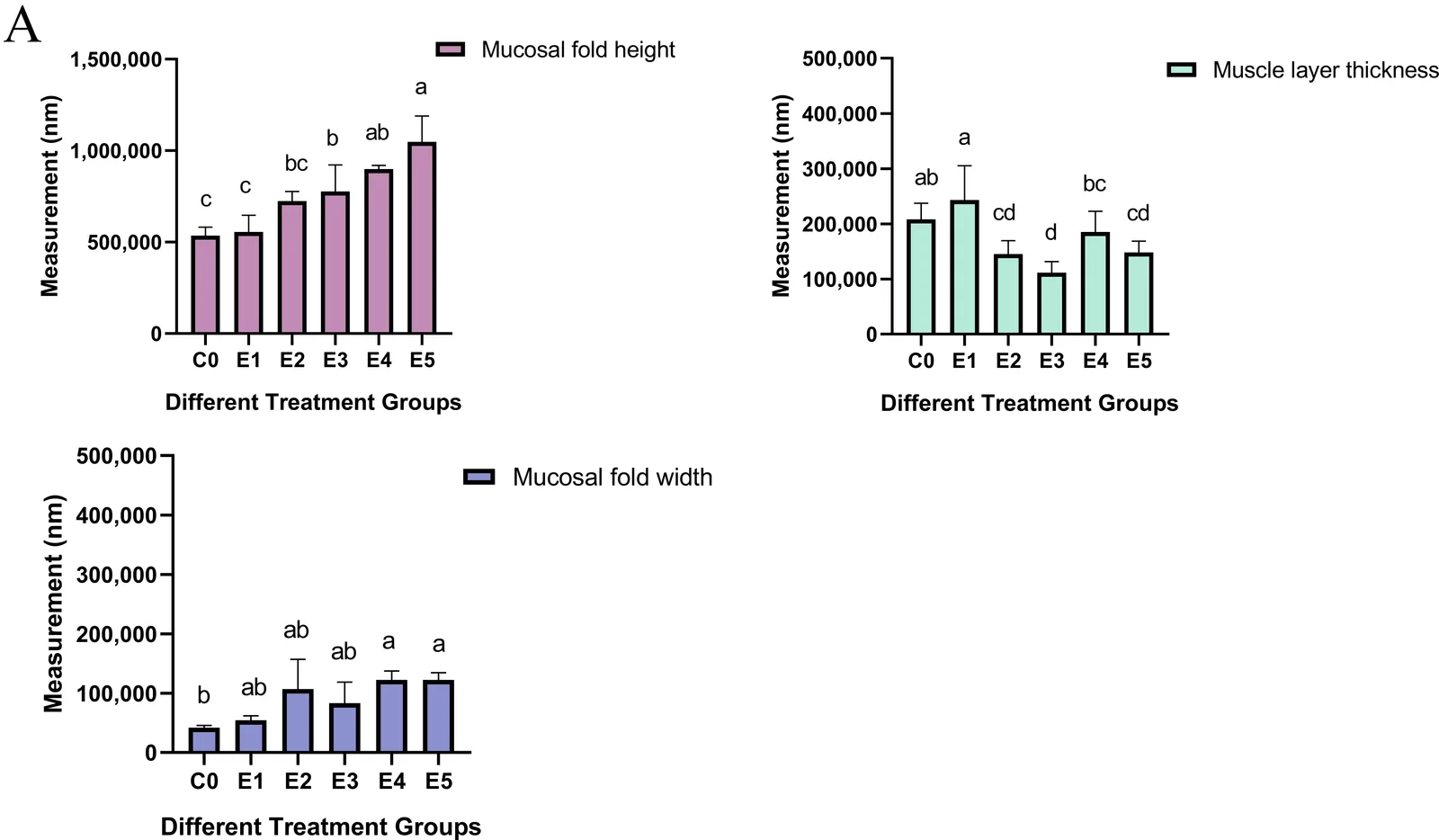

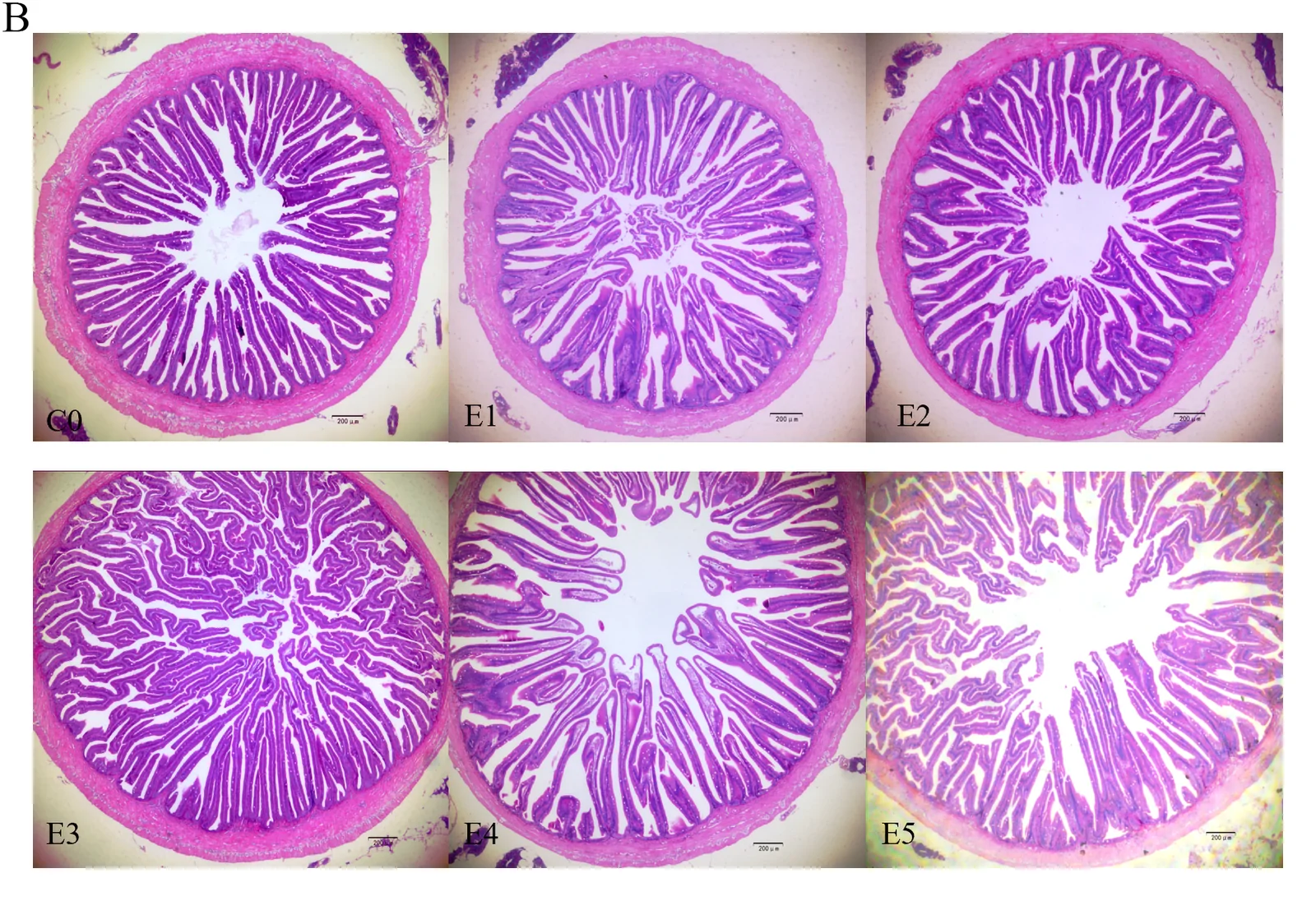
(**A**) Quantitative statistics of midgut morphological indexes (*n* = 6 per group); (**B**) representative histological sections of midgut tissue. Note: C0: control group; E1–E5: treatment groups with 1 × 10^5^ to 1 × 10^9^ CFU/g *E. faecalis* SZ07 supplementation. Scale bars = 200 µm. Different lowercase letters indicate significant differences at *p* < 0.05.

Changes in mid-intestinal morphological indices of golden pompano fed diets with graded levels of *E. faecalis* SZ07 are presented in Figure 2A. Relative to the C0 control group, mucosal fold height was markedly elevated in the E3, E4, and E5 treatment groups (*p* < 0.05). Mucosal fold width in the E4 and E5 groups was significantly greater than that in the control group (*p* < 0.05), whereas no statistically significant difference was found among the E1, E2, and E3 groups and the control. Regarding muscle layer thickness, significantly lower values were detected in the E2, E3, and E5 groups compared with the control group (*p* < 0.05).

Microscopic examination of intestinal sections showed that intestinal mucosal folds in the probiotic treatment groups were longer and arranged more compactly than those in the control group, along with reduced muscle layer thickness (Figure 2B). No histopathological lesions were detected in the intestinal tissues across all experimental groups. Collectively, these findings suggest that dietary *E. faecalis* SZ07 supplementation does not induce structural injury to the golden pompano intestine. Rather, it effectively enhanced intestinal absorption area and functional performance by improving mucosal fold morphological traits and decreasing muscle layer thickness.

### 3.2. Digestive, Antioxidant and Metabolic-Immune Enzyme Activities

**Figure 3:**
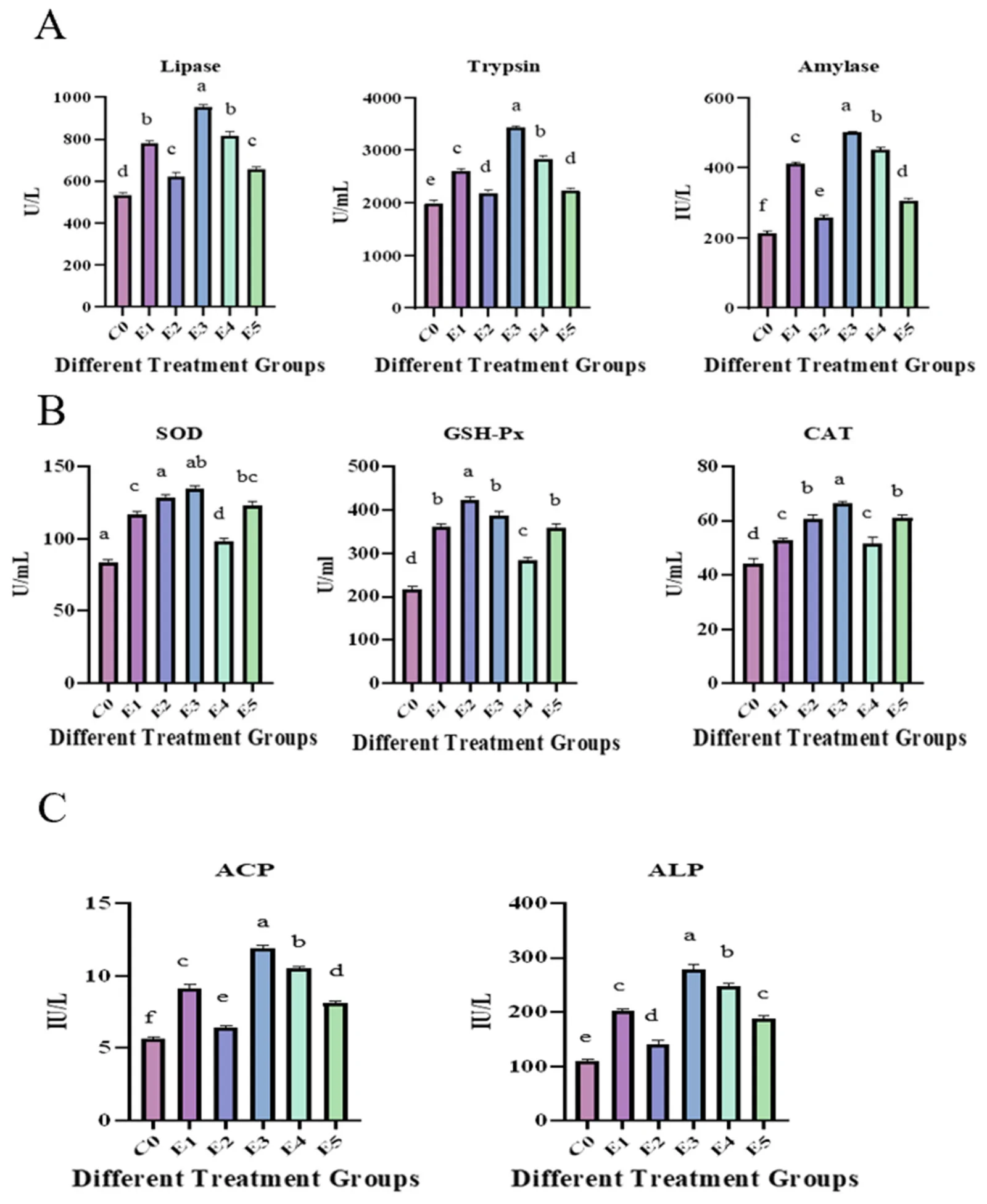
(**A**) Detection results of intestinal digestive enzyme activity; (**B**) detection results of antioxidant indicators in intestinal tissues; (**C**) detection results of metabolism-immunity bifunctional enzymes in intestinal tissues (*n* = 6 per group). Different lowercase letters indicate significant differences at *p* < 0.05.

Three core intestinal digestive enzymes of golden pompano were measured, and the corresponding data are shown in Figure 3A. One-way ANOVA revealed a significant effect of dietary SZ07 supplementation on lipase activity (*F* = 110.964, *p* < 0.001). Lipase activity was remarkably elevated in E1, E3, and E4 groups relative to the C0 control (*p* < 0.05), while no significant difference existed between E1 and E4 groups (*p* > 0.05). E2 and E5 also exhibited significantly higher lipase activity than C0. Similarly, trypsin activity rose significantly in E1, E3, and E4 treatments (*p* < 0.05), whereas E2 and E5 showed comparable trypsin levels to the control group. Amylase activity was significantly enhanced across all five probiotic supplementation groups compared with C0 (*p* < 0.05).

We further detected three key intestinal antioxidant enzymes, and relevant results are summarized in Figure 3B. SOD activity was significantly higher in E1, E4, and E5 groups than in the blank control (*p* < 0.05). Both GSH-Px and CAT activity were significantly elevated in all probiotic-supplemented groups (*p* < 0.05), and significant differences were observed between E2 and E4 for these two indicators.

Figure 3C illustrates how dietary SZ07 supplementation affects intestinal ACP and ALP activity. ACP activity was significantly enhanced in E3, E4, and E5 treatments compared with C0 (*p* < 0.05). Similarly, E3 and E4 groups showed remarkably higher ALP activity than the control (*p* < 0.05), while E1 and E5 exhibited ALP levels comparable to C0.

**Figure 4:**
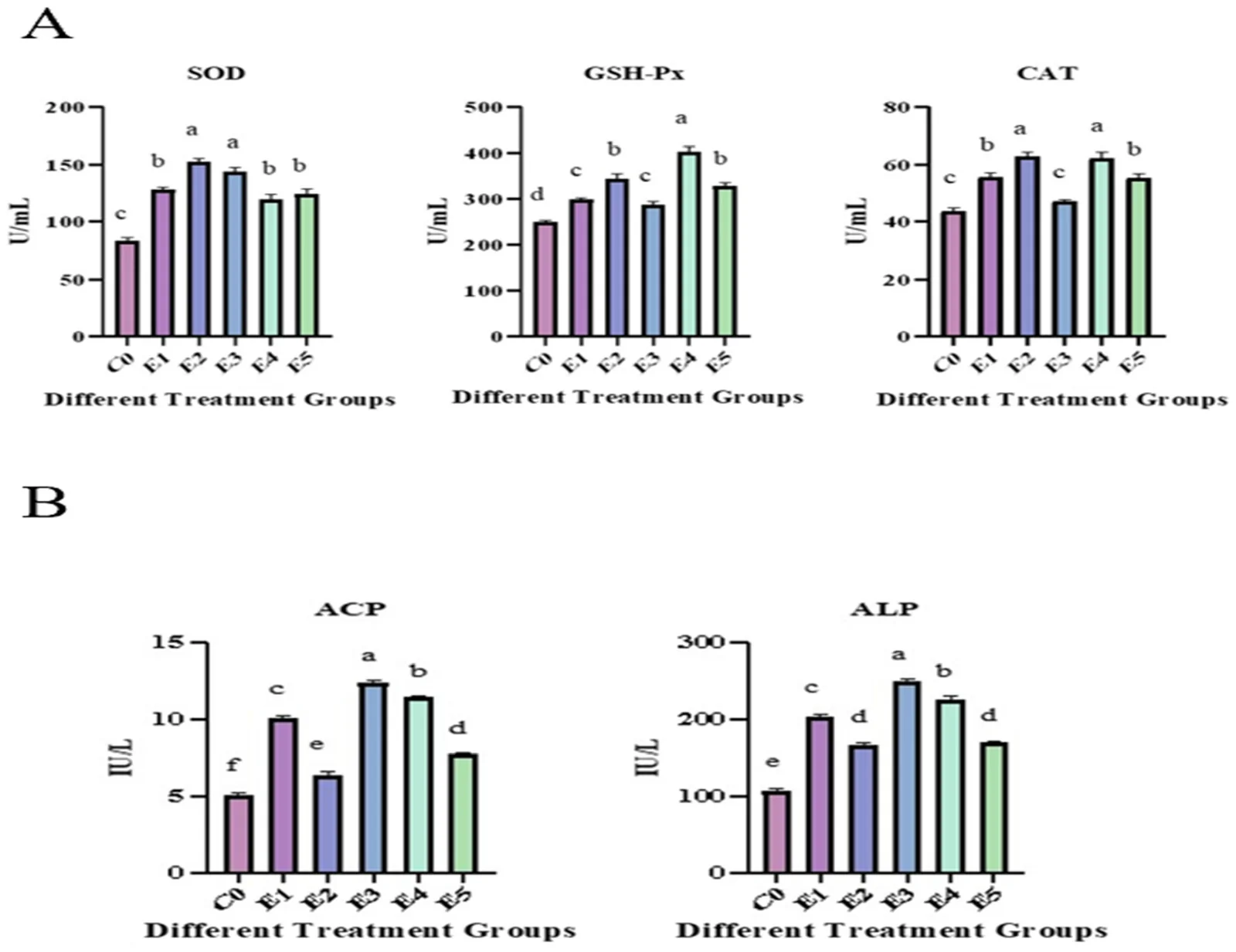
(**A**) Detection results of antioxidant indicators in the liver; (**B**) detection results of metabolism-immunity bifunctional enzymes in the liver (*n* = 6 per group). Different lowercase letters indicate significant differences at *p* < 0.05.

Figure 4A presents the measured antioxidant enzyme activity values in liver tissues. Compared with the blank C0 group, dietary SZ07 supplementation significantly increased hepatic SOD activity across all five treatment groups (*p* < 0.05). Comparable SOD levels were observed between E1, E4, and E5, as well as between E2 and E3 groups. Consistent with intestinal tissue results, hepatic GSH-Px activity was also markedly enhanced in all fish receiving SZ07-containing feeds (*p* < 0.05). No obvious inter-group differences in GSH-Px were found between E1 vs. E3 and E2 vs. E5. For hepatic CAT activity, E1, E2, E4, and E5 treatments showed remarkably higher activity than C0 (*p* < 0.05), while E3 displayed CAT levels comparable to the control group.

Figure 4B illustrates the hepatic activities of two immune-metabolic dual-function enzymes. Hepatic ACP activity was significantly boosted in E1, E2, E3, E4 and E5 groups relative to C0 (*p* < 0.05). For hepatic ALP, E1, E2, E3, E4, and E5 showed statistically elevated activity versus the control (*p* < 0.05).

### 3.3. Immune-Related Gene Expression in Spleen

**Figure 5:**
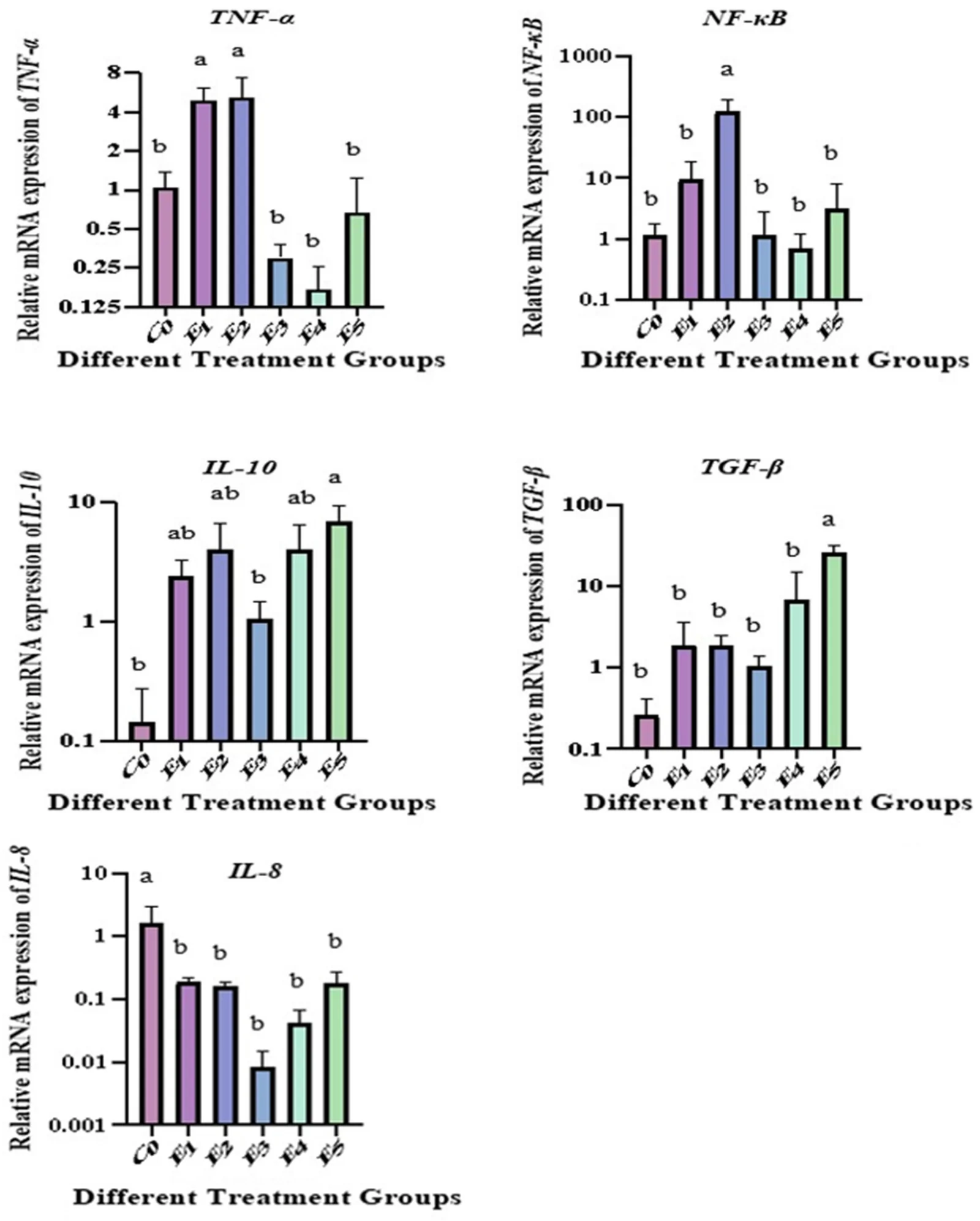
Real-time fluorescence quantitative PCR results of spleen (*n* = 6 per group). Note: Vertical axis shows mRNA transcript levels; abscissa labels show experimental groups. Different lowercase letters indicate significant differences at *p* < 0.05.

Figure 5 displays the expression patterns of immune-associated genes in the spleen. Dietary administration of graded doses of *E. faecalis* SZ07 exerted a marked regulatory effect on the splenic immune response of golden pompano.

Specifically, the mRNA level of TNF-α increased at low supplementary doses (E1 and E2) and decreased at moderate doses (E3 and E4). Relative to the C0 control group, *NF-κB* expression was significantly upregulated in the E2 group, whereas the remaining treatment groups exhibited irregular change trends. Conversely, the mRNA level of *IL-8* was significantly suppressed in all SZ07-supplemented groups relative to the C0 group (*p* < 0.05).

Notably, the E5 group had markedly higher *IL-10* and *TGF-β* mRNA levels than the control (*p* < 0.05). Although E1–E4 groups did not reach statistical significance for these two anti-inflammatory genes, their expression levels all rose to varying degrees against the C0 baseline.

### 3.4. Intestinal Microbiota Diversity

High-throughput sequencing of the V3–V4 hypervariable region of the 16S rRNA gene was performed on the Illumina MiSeq platform. After quality filtering and chimera removal, a total of 4730 ASVs were obtained from all samples.

We adopted 16S rRNA high-throughput sequencing to characterize the diversity and community composition of golden pompano intestinal microbiota, and full alpha diversity metrics are listed in Table 2. All samples achieved Good’s coverage above 0.99, which verifies sufficient sequencing depth to capture the majority of intestinal microbial taxa.

Analysis of alpha diversity revealed no statistically significant differences in the Chao1, Ace, Simpson, and Shannon indices across all experimental groups. Specifically, The Shannon index exhibited an upward trend in groups E3 and E4.

### 3.5. Intestinal Microbiota Structure

The PCoA ordination plot showed that the first two principal coordinates explained 3.62% and 3.45% of the total community variation, respectively. The distribution of samples in the PCoA coordinate space demonstrated that C0, E1, and E2 clustered closely along PCo1, while E3, E4, and E5 were widely separated along PCo2, indicating distinct microbial community reshaping effects under different SZ07 doses.

**Figure 6:**
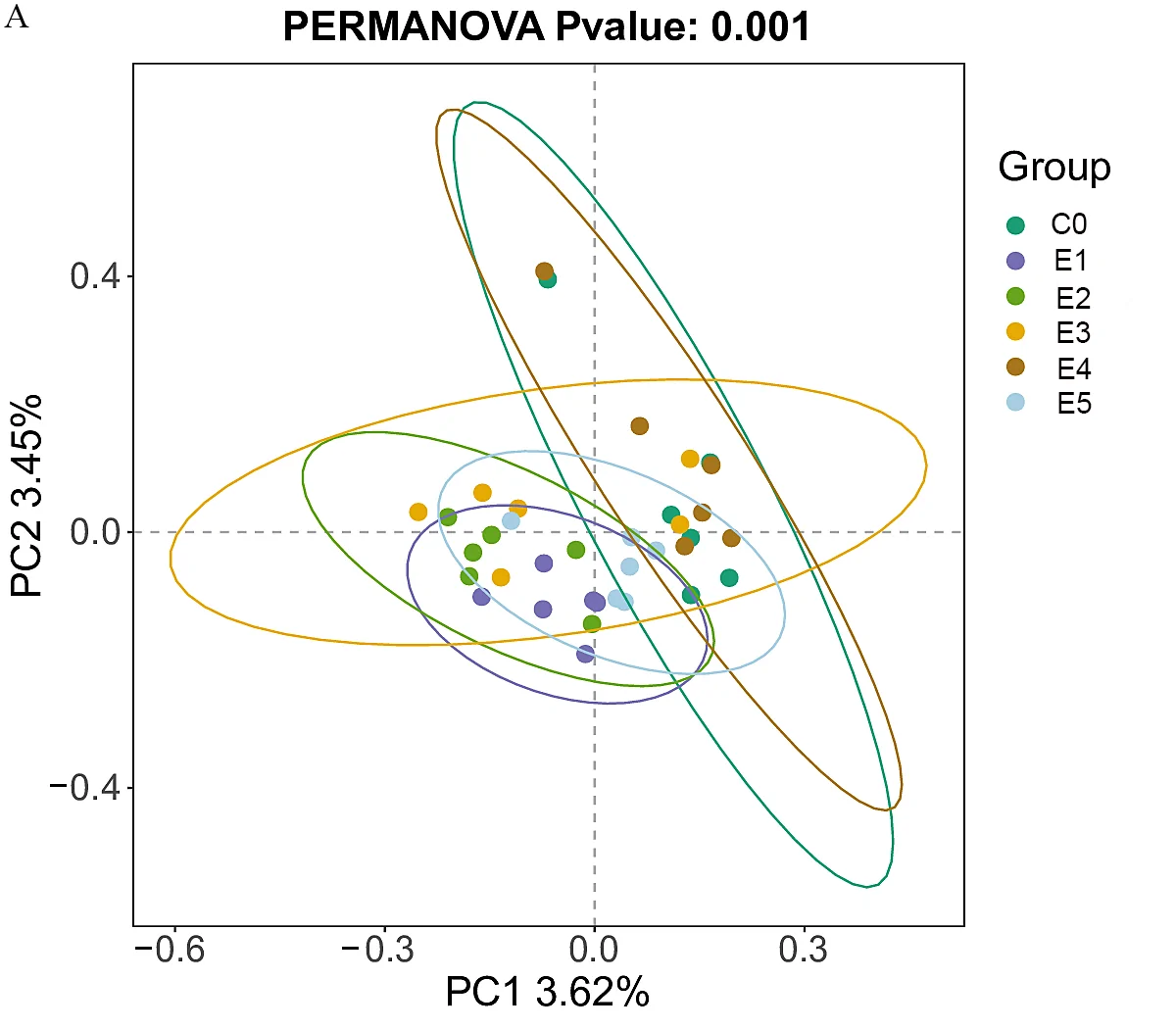

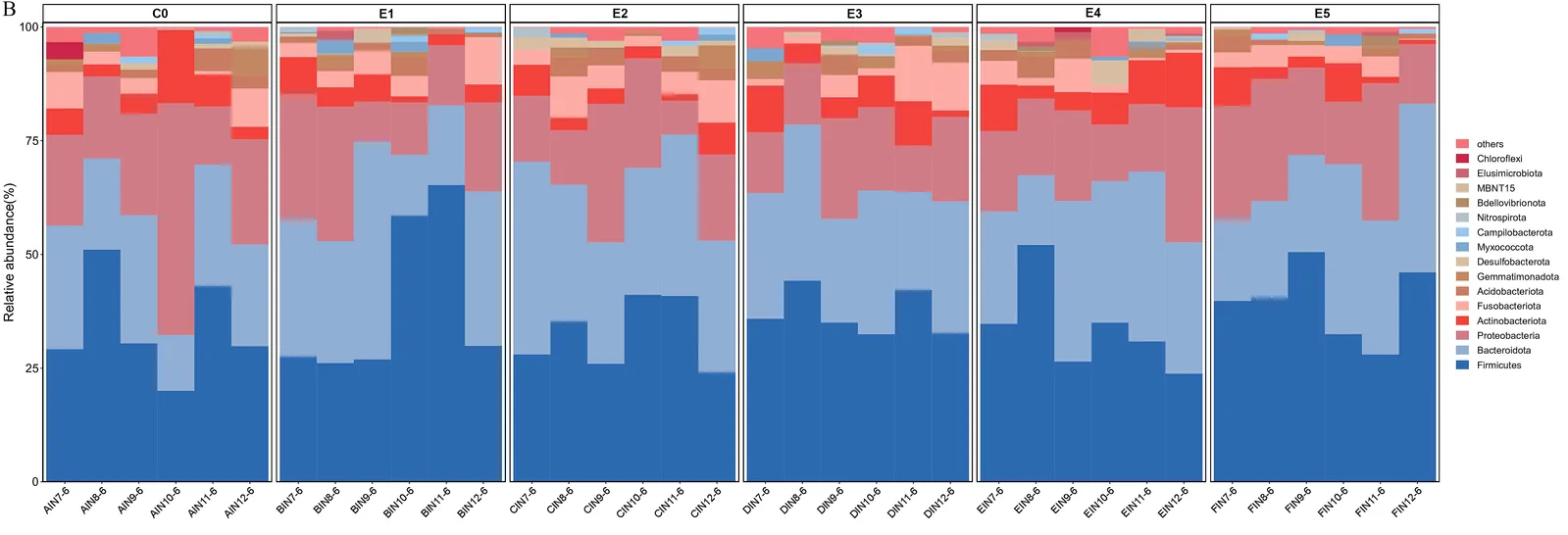

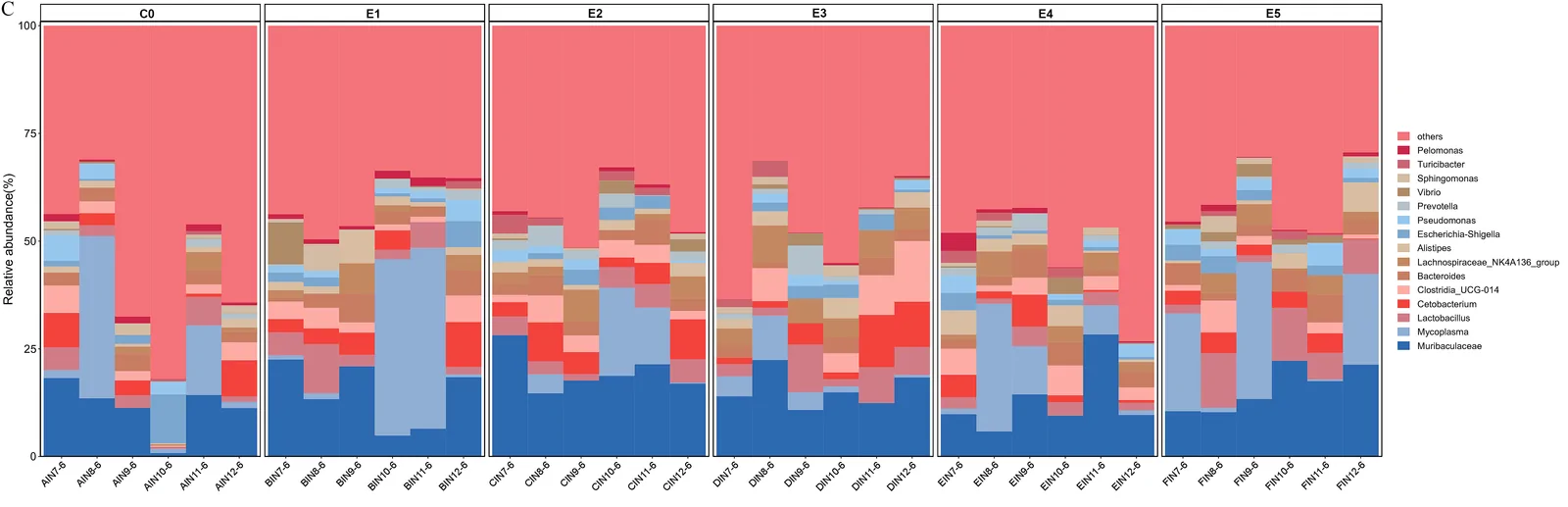
(**A**) PCoA (binary Jaccard distance) with PERMANOVA test; (**B**) top15 community composition bar plots (Phylum level); (**C**) top15 community composition bar plots (Genus level) (*n* = 6 per group) Note: C0: Add 0 CFU/g *Enterococcus faecalis* group; control group; E1: Add 1 × 10^5^ CFU/g *Enterococcus faecalis* group; E2: Add 1 × 10^6^ CFU/g *Enterococcus faecalis* group; E3: Add 1 × 10^7^ CFU/g *Enterococcus faecalis* group; E4: Add 1 × 10^8^ CFU/g *Enterococcus faecalis* group; E5: Add 1 × 10^9^ CFU/g *Enterococcus faecalis* group.

Figure 6B displays the taxonomic composition of gut microbiota at the phylum taxonomic rank. Eight dominant phyla were shared by all experimental groups: Proteobacteria, Firmicutes, Bacteroidetes, Actinobacteriota, Fusobacteriota, Acidobacteriota, Gemmatimonadota, and Desulfobacterota.

Genus-level gut microbial composition is visualized in Figure 6C. The dominant genera shared by all treatments included Muribaculaceae (family, CFB clade, order Bacteroidales), Mycoplasma, *Lactobacillus*, *Cetobacterium*, *Bacteroides*, and *Pseudomonas*. Compared with C0, *Lactobacillus* abundance tended to rise in all groups except E4. The relative proportion of *Cetobacterium* increased in E1–E3, while *Pseudomonas* abundance dropped to varying degrees within E1 to E4.

### 3.6. Differential Microbial Taxa Identified by Lefse Analysis

**Figure 7:**
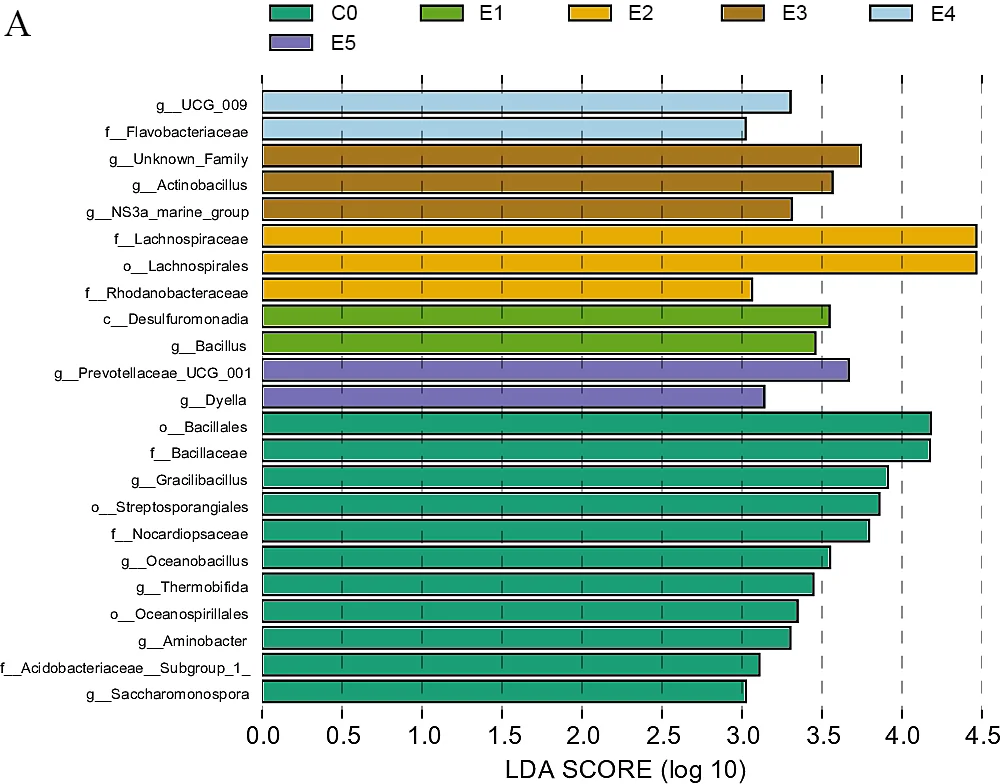

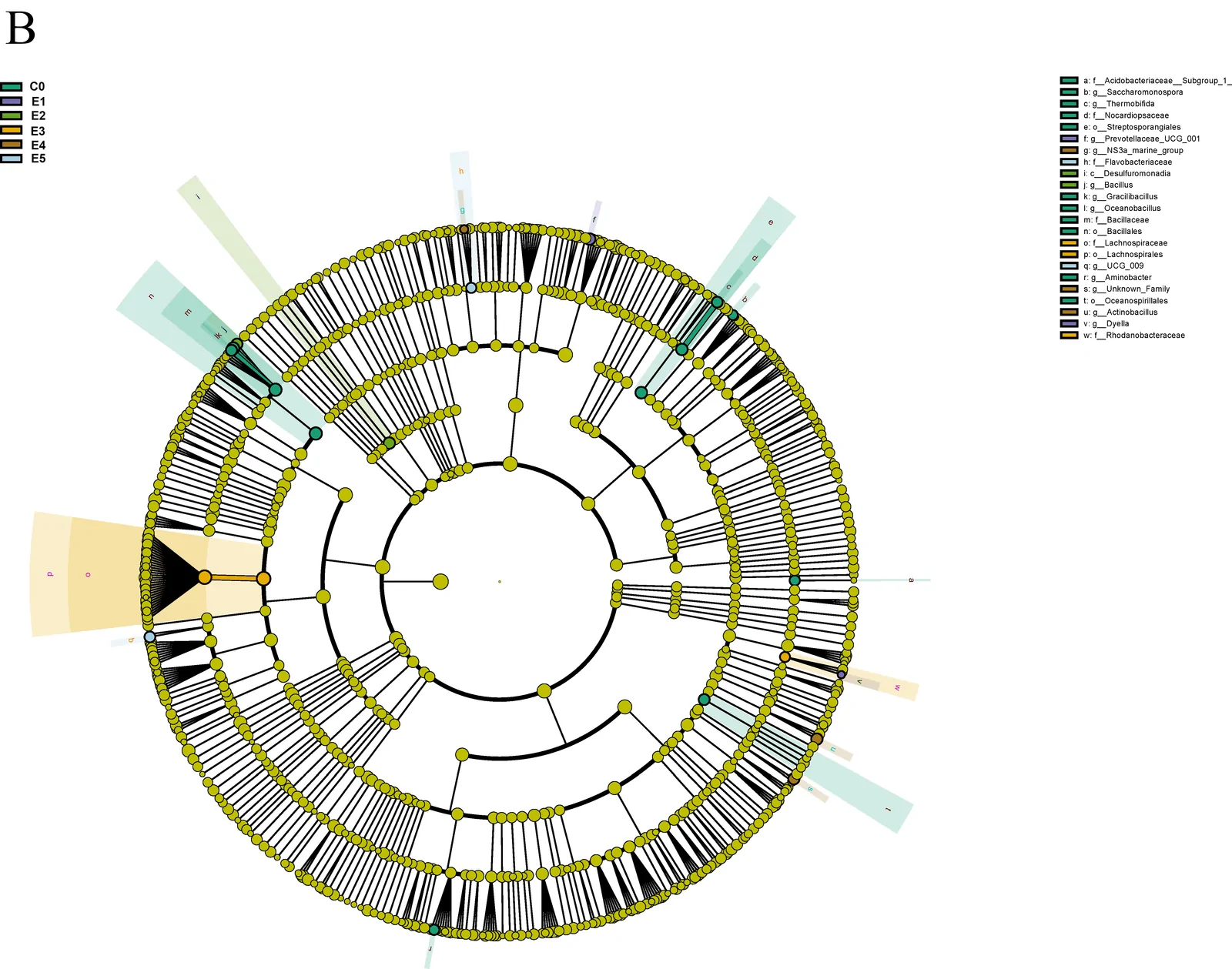
(**A**) Linear discriminant analysis (LDA) effect size plot; (**B**) branching diagram of differential species annotation (*n* = 6 per group). Note: Different colors indicate different groups; Each layer node represents the phylum/class/order/family/genus from the inside out. The annotations of species markers in each layer indicate phyla/class/order/family/genus from the inside out; The species names are shown in the legend on the right.

LEfSe (Linear Discriminant Analysis Effect Size) was applied to identify biomarker bacterial taxa with significantly differential abundance among treatments. Figure 7 contains the corresponding LDA score histogram and taxonomic evolutionary cladogram.

The results showed that the taxa significantly enriched in the control group (C0) included order Bacillales, family Bacillaceae, genera *OceanoBacillus*, *GraciliBacillus*, *Thermobifida*, *Saccharomonospora*, family Nocardiopsaceae, order Streptosporangiales, family Acidobacteriaceae__Subgroup_1_, order Oceanospirillales, and genus *Aminobacter*.

For the experimental groups, the characteristic differentially abundant taxa were as follows: Prevotellaceae_UCG_001 and *Dyella* were enriched in the E1 group; class Desulfuromonadia and genus *Bacillus* were enriched in the E2 group; order Lachnospirales and family Rhodanobacteraceae were enriched in the E3 group; NS3a_marine_group (unclassified order Bacteroidales) and genus *ActinoBacillus* were enriched in the E4 group; family Flavobacteriaceae and Ruminococcaceae_UCG_009 were enriched in the E5 group.

## 4. Discussion

### 4.1. Effect of E. faecalis SZ07 on Intestinal Morphology

*E. faecalis* has been extensively applied as a safe and efficacious probiotic in food supplements for both humans and animals, and has emerged as a promising antibiotic alternative in aquaculture for its capacity to improve fish health and mitigate disease outbreaks [26,27]. Notably, autochthonous *E. faecalis* strains isolated from the gut of host fish species exhibit better adaptability to the host intestinal microenvironment, with superior colonization capacity and higher biosafety compared with strains of terrestrial origin [28]. As a pivotal organ in fish, the intestine serves as both the primary site for digestion and nutrient uptake and the frontline barrier of innate immune defense. Sustaining intestinal homeostasis—including preserving mucosal fold structural integrity, regulating microbial balance, and boosting immune function—is essential for enhancing growth performance, disease resistance, and overall physiological health of farmed fish. Longer, more compact intestinal mucosal folds effectively expand the intestinal absorptive surface area, which further elevates the efficiency of nutrient utilization [29].

In this study, dietary administration of golden pompano-origin *E. faecalis* SZ07 exerted a notable dose-dependent influence on the intestinal morphological structure of golden pompano. Histological examination revealed that, relative to the C0 control group, mucosal fold height was significantly elevated in the E3, E4, and E5 groups (*p* < 0.05). Mucosal fold width in the E4 and E5 groups was significantly greater than that in the control group (*p* < 0.05), whereas no statistically significant difference was detected among the E1, E2, and E3 groups and the control. With respect to muscle layer thickness, significantly lower values were recorded in the E2, E3, and E5 groups in comparison with the control group (*p* < 0.05). The elevation of mucosal fold length and width enlarges the absorptive surface area of the intestinal mucosa, while the reduction in muscle layer thickness lowers energy expenditure for intestinal peristalsis. This allows more energy to be distributed to growth and immune function, which aligns with prior observations in Pacific white shrimp supplemented with *Clostridium butyricum* [30]. The reduction in intestinal muscular layer thickness may reflect tissue remodeling of the gut wall. Altered muscular thickness can modify intestinal peristalsis and digesta transit, although it may also represent structural atrophy under certain conditions. Further functional assays are required to distinguish these possibilities [31]. Overall, these morphological alterations offer direct histological proof that *E. faecalis* SZ07 can enhance intestinal health and nutrient absorption capacity in golden pompano.

### 4.2. Modulation of Intestinal Digestive Enzyme Activities

Digestive enzymes play a crucial role in the digestion and nutrient absorption process of aquatic animals [32]. These enzymes, secreted by the fish itself or produced by intestinal microbiota, decompose macronutrients such as proteins, fats, and carbohydrates into small absorbable molecules including amino acids, fatty acids, and monosaccharides. *E. faecalis* as a feed additive has been widely reported to enhance intestinal digestive enzyme activity, thereby improving intestinal health and innate immunity of fish [33]. Accumulating evidence confirms that *E. faecalis* is capable of secreting multiple digestive enzymes, including amylase and protease; these enzymes directly enhance the catabolic function of the fish digestive tract and boost feed utilization efficiency [34].

Our current findings demonstrate that dietary administration of golden pompano-origin *E. faecalis* SZ07 markedly increased intestinal digestive enzyme activities in golden pompano. Specifically, amylase activity was significantly upregulated in all treatment groups relative to the C0 control group (*p* < 0.05). The activities of both lipase and trypsin were significantly enhanced in the E1, E3, and E4 groups compared with the C0 group (*p* < 0.05), whereas no statistically significant changes were detected in the E2 and E5 groups. The uniform and significant elevation of amylase activity in all SZ07-supplemented groups is especially remarkable, which corresponds to the intrinsic property of *E. faecalis* to synthesize large amounts of amylase [34]. These findings suggest that dietary supplementation of *E. faecalis* SZ07 at suitable dosages can effectively strengthen the digestive enzyme activities of golden pompano, thereby improving its nutrient digestion and absorption capacity. As an autochthonous probiotic strain isolated from healthy golden pompano, SZ07 exhibits better adaptability to the intestinal microenvironment of its host species, which may account for its stable and robust digestion-promoting efficacy.

### 4.3. Enhancement of Antioxidant and Non-Specific Immune Functions

The improved digestive function further promoted the elevation of antioxidant capacity and non-specific immune function in golden pompano [35]. The antioxidant enzyme system serves as a critical defense system in living organisms; it eliminates excessive reactive oxygen species (ROS) and free radicals to shield cells from oxidative injury [36]. As core components of the antioxidant defense system, superoxide dismutase (SOD), glutathione peroxidase (GSH-Px), and catalase (CAT) activities are widely adopted as biomarkers to assess fish health condition and antioxidant capacity [37]. During aquaculture farming, fish are vulnerable to various environmental stressors, including temperature fluctuations, water quality degradation, and pathogen infection, all of which trigger excessive ROS generation. Strengthening the antioxidant enzyme system assists fish in counteracting oxidative stress and boosting immune competence [38].

Our results revealed that dietary *E. faecalis* SZ07 supplementation markedly elevated antioxidant enzyme activities in golden pompano. Specifically, significantly higher activities of SOD, GSH-Px, and CAT in both liver and intestine were detected in the E4 and E5 groups relative to the C0 group (*p* < 0.05). In the E3 group, hepatic and intestinal GSH-Px activity, hepatic SOD activity, and intestinal CAT activity were all significantly increased compared with the control group (*p* < 0.05), whereas no significant changes were found for the other indices. Prior research has indicated that dietary *E. faecalis* supplementation can boost antioxidant enzyme activities in mud crabs, thus modulating the immune system and enhancing disease resistance [39]. Another study reported that *E. faecalis*, when used as a feed additive, can significantly raise SOD activity in sea cucumbers and benefit their growth performance [40].

Acid phosphatase (ACP) and alkaline phosphatase (ALP) are two important phosphatases widely present in organisms and involved in various physiological processes [41,42]. Their activities in fish are often used as biomarkers to evaluate health status, metabolic function, and immune response, and they are mainly distributed in tissues such as the liver and intestine [43]. ACP mainly reflects immune status, while ALP mainly reflects growth and metabolic status [42,44].

Notably, the activities of both ACP and ALP exhibited a marked upward trend in all E1–E5 treatment groups relative to the C0 group (*p* < 0.05). Previous studies have demonstrated that *E. faecalis* and *Lactococcus lactis*, applied individually or in combination as candidate probiotics, can elevate ACP and ALP activities, thus strengthening humoral immunity and boosting disease resistance [45].

The improvement of non-specific immune capacity was also mirrored by the altered expression of immune-associated genes in the spleen. Probiotics are able to interact with intestinal immune cells and commensal microbiota to regulate targeted immune functions and preserve immune homeostasis. Cytokines are involved in a wide range of physiological processes, such as the modulation of immune and inflammatory responses [46,47]. *TNF-α* and *IL-8* are representative pro-inflammatory cytokines that perform diverse functions in initiating inflammatory responses [48]. Upregulated *NF-κB* expression is regarded as a critical pathogenic contributor to various acute and chronic inflammatory disorders [49].

In the present study, dietary administration of *E. faecalis* SZ07 at suitable concentrations markedly suppressed the mRNA expression levels of *TNF-α* and *IL-8* in the spleen of golden pompano. The expression pattern of *NF-κB* displayed irregular changes across the experimental groups relative to the control group. *IL-10* and *TGF-β* are anti-inflammatory cytokines capable of inhibiting excessive inflammatory reactions and sustaining immune equilibrium [50]. Significantly higher expression levels of *IL-10* and *TGF-β* were observed in the E5 group compared with the control group (*p* < 0.05). Despite the absence of statistically significant differences in the E1–E4 groups, both genes exhibited upward trends to different extents relative to the C0 group. These findings suggest that dietary *E. faecalis* SZ07 can alleviate tissue inflammatory reactions via upregulating the expression of anti-inflammatory cytokine genes. It should be noted that this experiment was carried out without pathogen challenge. The inflammatory cytokine response induced by this strain varied with dosage, showing immune priming at low doses and an anti-inflammatory tendency only at the highest dose [4]. It has been proposed that probiotic-derived metabolites such as short-chain fatty acids may activate the Nrf2 signaling pathway to modulate the antioxidant system in golden pompano. It should also be noted that increased antioxidant enzyme activity may not always be beneficial, and such elevation can occasionally serve as a compensatory response to oxidative stress [51].

### 4.4. Regulation of Intestinal Microbiota Structure

Stable intestinal immune homeostasis largely relies on the structural balance of host gut microbial communities [52]. As a safe and effective feed supplement, probiotics can markedly improve the health condition and growth performance of farmed animals by optimizing intestinal microecological balance, boosting immune function, and facilitating nutrient absorption [53].

Alpha diversity analysis revealed no statistically significant differences in the Chao1, Ace, Simpson, and Shannon indices across all groups. Nevertheless, the Shannon index exhibited an upward trend in the E3 and E4 groups relative to the C0 group, indicating that moderate doses of *E. faecalis* SZ07 may exert a beneficial effect on gut microbiota evenness. Prior research has shown that oral probiotic supplementation elevated the relative abundance of beneficial bacteria and decreased the proportion of pathogenic bacteria in the shrimp intestine [54]. Another study reported that probiotic intervention at an appropriate dosage can effectively enhance the species richness and evenness of gut microbiota in *Litopenaeus vannamei* [55]. These results suggest that *E. faecalis* may modulate the composition of indigenous microbiota through competitive exclusion, and appropriate concentrations of *E. faecalis* can improve the community distribution evenness of intestinal microbiota in golden pompano [56].

Consistent with the trend of improved microbiota evenness, the overall structure of the intestinal microbiota was modulated by dietary *Enterococcus faecalis* SZ07. In most teleost fish, Proteobacteria, Firmicutes, and Bacteroidetes are the dominant phyla in the intestinal microbiota, which play crucial roles in host digestion, immunity, and overall health [57]. Members of Proteobacteria have diverse metabolic capabilities and can utilize various carbon and nitrogen sources. Many bacteria in Firmicutes, such as *Bacillus*, are widely used as probiotics to improve fish intestinal health. Bacteroidetes bacteria can modulate host immune reactions and balance the release of pro-inflammatory and anti-inflammatory cytokines to avoid excessive inflammatory responses [58,59,60].

Our results verified that these three phyla constituted the dominant bacterial communities in the intestinal microbiota of golden pompano across all groups. Relative to the C0 control group, dietary *E. faecalis* SZ07 altered the proportional distribution of Proteobacteria, Firmicutes, and Bacteroidetes, suggesting that it did not shift the core dominant-phylum composition of the golden pompano gut microbiota. At the genus taxonomic level, *Lactobacillus* and *Cetobacterium* represented the predominant genera across all groups. As a typical lactic acid bacterial genus, *Lactobacillus* is frequently employed as a probiotic to ameliorate digestive health and strengthen immune function [11]. As a dominant symbiotic bacterium colonizing the fish intestine, *Cetobacterium* is prevalent across diverse fish species and exerts a potential function in preserving fish health via modulating gut microbiota structure and metabolic performance [61].

In agreement with the above results, *Lactobacillus* abundance was elevated in all treatment groups with the exception of the E4 group relative to the control group. *Cetobacterium* abundance was raised in the E1, E2, and E3 groups, whereas *Pseudomonas* abundance was reduced in the E1, E2, E3, and E4 groups. Certain *Pseudomonas* species, exemplified by *Pseudomonas aeruginosa*, are opportunistic pathogens that trigger infections when aquatic animals have compromised immunity [62]. These results indicate that dietary supplementation with appropriate amounts of *E. faecalis* SZ07 can improve host intestinal health through a dual mechanism: on the one hand, it significantly enhances the digestive and absorptive capacity and immune function of golden pompano; on the other hand, it effectively regulates the intestinal microbiota structure, manifested as a tendency for reduced abundance of potential pathogens (such as *Pseudomonas*) and increased colonization of beneficial bacteria (such as *Lactobacillus*), ultimately improving intestinal health and overall host health status. Notably, the genus *Mycoplasma* was identified as a dominant taxon in the intestinal microbiota of golden pompano. Consistent with previous reports on golden pompano, this genus represents a core host-associated intestinal component rather than a sequencing contaminant [63]. Host-adapted *Mycoplasma* are frequently detected as dominant commensals in the gut of marine teleosts, and some strains exhibit mutualistic interactions with fish hosts [64].

LEfSe screening was further performed to screen distinct biomarker taxa with differential abundance across all treatments, which provided more detailed information about the modulation of intestinal microbiota by *E. faecalis* SZ07. The characteristic taxa in the E5 group were Flavobacteriaceae and Ruminococcaceae_UCG_009. Flavobacteriaceae are widely distributed in marine environments and can degrade complex organic matter such as proteins and polysaccharides, participating in carbon and nitrogen cycles. Ruminococcaceae can ferment complex polysaccharides such as cellulose and starch to produce short-chain fatty acids (SCFAs), which help maintain intestinal health [65,66]. The characteristic taxa in the E4 group were NS3a_marine_group (unclassified Bacteroidales) and *ActinoBacillus*. Bacteria of the order Bacteroidales may participate in the degradation of organic matter and nutrient cycling in marine ecosystems [67].

The characteristic taxa in the E3 group were Lachnospirales and Rhodanobacteraceae. Most members of Lachnospirales are anaerobic bacteria with fermentative and chemoorganotrophic capabilities [68]. Rhodanobacteraceae are widely distributed in aquatic environments such as rivers, lakes, and oceans, and are common in eutrophic waters [69]. The characteristic taxa in the E2 group were Desulfuromonadia and *Bacillus*. *Bacillus* bacteria can form endospores and have strong environmental adaptability. In aquaculture, *Bacillus* species are often introduced as probiotics to improve water quality and promote the health of aquatic animals. Accumulating evidence has demonstrated that *Bacillus* can elevate digestive enzyme activities, activate antioxidant enzyme systems, and upregulate the expression of immune- and stress-associated genes, thus enhancing growth performance and disease resistance in farmed fish [11]. Accordingly, the increased abundance of *Bacillus* in the E2 group may have exerted a synergistic effect on the observed health-promoting benefits. The observed enrichment of *Bacillus* in the E2 group cannot be definitively explained by the present dataset. This phenomenon may reflect cross-feeding or niche modification driven by metabolites produced by *E. faecalis*. Further co-culture assays or in vivo animal trials are required to uncover the underlying inter-microbial interaction.

### 4.5. Limitations of the Study

The present study is subject to several notable limitations. First, this feeding trial was performed in large-capacity floating net cages, which were selected to match the natural schooling and continuous swimming behavior of golden pompano. Research shows that limited rearing space can induce severe confinement-related stress in fish and impair their physiological and immune functions, which is critical for evaluating the biological effects of dietary probiotic supplementation [2,70]. Restricted by the layout of the commercial aquaculture facility, each dietary group was maintained within one large floating cage unit, and biological replicates were obtained from individual fish sampled within the corresponding unit. 

Second, the present experiment evaluated the short-term effects of dietary probiotics on juvenile golden pompano under relatively stable water quality conditions. It is worth noting that offshore cage culture environments are highly dynamic, where temperature, salinity, and dissolved oxygen fluctuate frequently under natural weather conditions. Based on this, we believe that the physiological and immune responses of golden pompano to probiotic diets may differ substantially under variable field conditions. These confounding factors may limit the direct extrapolation of the present findings to full-scale commercial marine cage farming, and the practical applicability of these probiotic formulations therefore remains to be further validated.

In the interpretation of the present study’s findings, it is critical to account for the fact that the probiotic effects were only assessed at the physiological and immune level of juvenile fish. While this study identified the beneficial effects of dietary probiotics on fish physiological performance, the long-term persistence of probiotic colonization in the gut and the sustained regulatory effects on intestinal microbiota were not fully quantified during the experimental period. That said, all experimental fish were reared in identical large-capacity cages and exposed to consistent background environmental conditions throughout the trial, thereby ensuring their uniform exposure to the probiotic-supplemented diets. On this basis, we believe that the observed differences between dietary groups are mainly attributable to dietary probiotic treatments rather than background environmental disturbance.

In addition, viable bacterial enumeration was only performed for the initial liquid bacterial suspension, and we did not re-quantify viable counts in experimental diets after spraying, drying, and storage. Background loads of *Enterococcus* and lactic acid bacteria in the basal commercial feed were also not analyzed, which may bring uncertainties to the actual effective dosage of probiotic in feeding practice. Moreover, feed consumption per cage was not quantified in this trial, which restricts our ability to assess feed efficiency and nutrient utilization among treatments. Nevertheless, fish were fed to apparent satiation twice daily under identical feeding protocols across all groups to minimize potential feeding bias. Although each dietary treatment was allocated to a single floating cage under commercial farm constraints and growth and survival data were not recorded, the consistent physiological and immunological responses across measured indices provide preliminary support for the bioactivity of SZ07, as observed in similar dose-screening aquaculture trials [71]. The present findings offer preliminary field-relevant information regarding the suitable dose range of dietary *E. faecalis* SZ07 for golden pompano. Future work should validate these outcomes using multiple independent cage replicates per treatment, and further clarify the long-term gut colonization dynamics, microbiota succession, and stress resistance benefits of SZ07 under fluctuating offshore cage conditions.

The deficiencies in this measurement provide implications for future research. Besides extending the feeding cycle to track long-term probiotic regulation on gut microbiota and immune homeostasis, expanding the experimental scale and optimizing the treatment design of target fish are also recommended. Additional studies can also carry out systematic assessments of probiotic stability in feed under farm conditions, host gut microbial succession, and the synergistic interactions between probiotics and environmental stressors, so as to improve the validity and applicability of probiotic feeding strategies for golden pompano industrial culture.

## 5. Conclusions

This study demonstrated that host-derived *E. faecalis* SZ07 serves as a promising and efficient feed additive for juvenile golden pompano culture. Appropriate dietary supplementation with SZ07 positively regulated intestinal morphological structure by increasing mucosal fold height and width while reducing muscular layer thickness, which effectively improved the intestinal digestive and absorptive capacity of juvenile golden pompano. Meanwhile, SZ07 supplementation significantly enhanced the activities of digestive enzymes, antioxidant enzymes, and immune-related metabolic enzymes, and balanced the intestinal immune response by downregulating pro-inflammatory gene expression and upregulating anti-inflammatory gene levels. Furthermore, dietary SZ07 optimized intestinal microbiota composition, enriched beneficial microbial communities, and inhibited the proliferation of potential pathogenic bacteria. Notably, the supplementation dose of 1 × 10^7^–1 × 10^8^ CFU/g feed exhibited the most stable and comprehensive beneficial effects among all treatment groups. From an economic perspective for on-farm production, 1 × 10^7^ CFU/g feed (E3) is the preferred option for commercial use, as elevating the dosage to 1 × 10^8^ CFU/g feed brings limited additional benefits but increases the input cost of probiotics.

Although one net-cage was assigned to each dietary treatment in this trial, limiting broad statistical extrapolation, the consistent intestinal, biochemical, and microbial responses among sampled fish support the credibility of our findings. Benefiting from the large-capacity net-cage culture system adopted in the present study, sufficient swimming space effectively reduced confinement stress interference, ensuring that the physiological and intestinal responses of golden pompano were mainly attributed to dietary probiotic intervention rather than environmental disturbance. Based on these findings, host-derived *E. faecalis* SZ07 has been verified to effectively improve intestinal health, antioxidant capacity, and immune homeostasis of golden pompano under aquaculture conditions, showing great application potential as a safe and sustainable green feed additive in mariculture.

Future studies could further explore the long-term colonization stability of SZ07 in the intestinal tract and verify its persistent regulatory effects on growth performance and disease resistance under complex commercial aquaculture conditions. In conclusion, dietary supplementation with host-derived *E. faecalis* SZ07 is an effective and feasible strategy to improve the overall health status of golden pompano, which provides a reliable theoretical basis and technical reference for the healthy and sustainable culture of golden pompano.

## Figures and Tables

**Table 1:** Primer sequences.

Gene	Primer Sequence (From 5′ to 3′)
*TNF-α*-F	CGCAATCGTAAAGAGTCCCA
*TNF-α*-R	AAGTCACAGTCGGCGAAATG
*NF-κB*-F	TGCGACAAAGTCCAGAAAGAT
*NF-κB*-R	CTGAGGGTGGTAGGTGAAGGG
*IL-8*-F	GAGAAGCCTGGGAATGGA
*IL-8*-R	GAGCCTCAGGGTCTAAGCA
*IL-10*-F	CGTCCTGGCTCTCTTGTCTCTCCTC
*IL-10*-R	TGTCCATGTCATTGTTTGCCTCATA
*TGF-β*-F	TATCCCTCTACAACAGCACCA
*TGF-β*-R	GGTCAGCAGGCGGTAATC
*β-actin*-F	TACGAGCTGCCTGACGGACA
*β-actin*-R	GGCTGTGATCTCCTTCTGC

**Table 2:** Analysis of gut microbiota abundance and diversity (*n* = 6 per group).

	Treatments					
Parameter	C0	E1	E2	E3	E4	E5
PD_whole_tree	21.63 ± 9.48	18.78 ± 2.91	18.16 ± 1.28	19.28 ± 1.69	21.61 ± 8.05	18.24 ± 1.48
Chao1	207.03 ± 165.75	149.73 ± 25.23	145.59 ± 15.07	150.90 ± 20.43	195.57 ± 123.57	144.95 ± 10.95
Shannon	6.12 ± 0.66	5.83 ± 0.93	6.10 ± 0.38	6.31 ± 0.26	6.42 ± 0.92	5.99 ± 0.36
Simpson	0.96 ± 0.05	0.93 ± 0.08	0.97 ± 0.01	0.98 ± 0.01	0.97 ± 0.03	0.96 ± 0.03
Goods_coverage	0.99 ± 0.0000116	0.99 ± 0.0000205	0.99 ± 0.0000146	0.99 ± 0.0000138	0.99 ± 0.0000159	0.99 ± 0.0000131
Ace	209.06 ± 164.86	151.35 ± 24.84	146.31 ± 14.52	152.45 ± 20.29	197.28 ± 124.48	145.49 ± 10.86

## Data Availability

The original contributions presented in this study are included in the article. Further inquiries can be directed to the corresponding author(s).
